# Antimicrobial coating is associated with significantly lower aerobic colony counts in high-touch areas in an orthopedic ward environment

**DOI:** 10.1186/s12941-020-00406-7

**Published:** 2020-12-14

**Authors:** Lars Ejerhed, Leyla Roshani, Annette Erichsen Andersson

**Affiliations:** 1 Department of Research and Development, NU-Hospital Group, Trollhättan, Sweden; 2 Institute of Clinical Sciences, Sahlgrenska Academy, University of Gothenburg, Gothenburg, Sweden; 3 Institute of Health and Care Sciences Sahlgrenska Academy, University of Gothenburg, Gothenburg, Sweden; 4 Department of Orthopedics, Sahlgrenska University Hospital, Gothenburg, Sweden

**Keywords:** Antimicrobial surface coating, Organosilane, High touch areas, Aerobic colony count (ACC), Hospital acquired infections (HAI)

## Abstract

**Background:**

Hospital acquired infections (HAI) are the most common complication found in the hospital environment. The aim of the study was to examine whether the use of an antimicrobial coating in high-touch areas in an orthopedic ward could reduce bacterial growth and HAI.

**Methods:**

From December 2017 to February 2018, HAI were registered on two orthopedic wards. A second registration was performed from December 2018 to February 2019. On the second occasion, an antimicrobial organosilane coating was applied just before the study period and thereafter weekly on one ward, while the other ward served as a control. Twenty defined high-touch areas on each ward were cultured before treatment and after 1, 2, 4, 8, 12, 14 and 16 weeks. Samples were cultured for aerobic colony counts, *Staphylococcus aureus* and *E. coli.*

**Results:**

The total aerobic colony counts were 47% lower on the treated ward compared with the non-treated ward over the study period (p = 0.02). The colony counts for *Staphylococcus aureus* and *E. coli* were low on both wards. During the first registration period, the incidence of HAI was 22.7% and 20.0% on the non-treated and subsequently treated ward respectively. On the second occasion, after treatment, the incidence was 25.0% and 12.5% (treated ward) respectively (p = 0.0001).

**Conclusions:**

The use of a long-lasting antimicrobial organosilane coating appears to reduce the bioburden and reduce HAI. Since the incidence of HAI varies substantially over time, longer observation times are needed.

## Introduction

Hospital acquired infections (HAI) are the most common complication found in the hospital environment and they result in significant patient morbidity and mortality. Despite careful hygiene routines and the more restrictive use of antibiotics, there is an increasing problem with serious infections and resistance to antibiotics.

It has been proposed that an increased environmental bioburden on a ward could result in an increased HAI risk [1, 2]. The optimal manual cleaning process is yet to be found. Most cleaning methods and disinfectants have a good immediate effect, but the cleaned surfaces are quickly recontaminated [3, 4]. One important question is whether the current cleaning routines/disinfectants can be supplemented in order to reduce the bioburden. Antimicrobial surfaces in the form of metals, such as silver and copper, have been investigated for many years. In clinical trials, copper surfaces have been shown to lower the concentrations of bacteria and the rate of HAI [2, 5–7]. Commonly used disinfectants, such as chlorine, hydrogen peroxide and alcohol, have no residual effect after drying and the treated surface can be recontaminated within minutes. Self-disinfecting surfaces that act against microbes could therefore be an interesting option. Tamimi et al. demonstrated long-term efficacy using a quaternary ammonium organosilane compound, reducing the total number of bacteria for 15 weeks [8].

The high-touch areas on a hospital ward are of special interest, as they could serve as a contamination source.

The aim of the present study was to examine whether the use of an organosilane antimicrobial coating without a quaternary ammonium compound on an orthopedic ward could reduce bacterial growth and HAI. The hypothesis of the study was that, by adding an organosilane compound to the usual cleaning routines, the number of bacteria could be halved.

## Materials and methods

### Study environment

The study was conducted on two 26-bed orthopedic wards in a community hospital in Western Sweden. The wards were each other’s mirror images, with a similar staff and patient mix. The treated ward was an acute orthopedic geriatric (AOG) ward, while an acute orthopedic (AO) ward served as a control. The patients were mainly suffering from fractures, pain problems, infections and threatening ischemic conditions.

The antimicrobial surface coating, Bioshield® 75, that was used is manufactured by Novalent, Greensboro, NC, USA. Bioshield® 75 is an aqueous organosilane. According to the manufacturer, it is a long-acting antimicrobial biostatic surface protectant and it has a mechanical and galvanic mode of action, resulting in the disruption of the cellular function and subsequent cellular death (https://novalent.com/technology/products/). The actual durability of the surface coating depends on type of substrate used for wiping and the level of abrasion the surface would see under normal use between wipings. For high touch surfaces in hospital environments, the manufacturer expects it to last one week at a minimum, and begin to degrade over the following two weeks. High touch areas cleaned with alcohol are therefore recommended for Bioshield® 75 touch up on a weekly basis (personal communication with the manufacturer).

**Fig. 1:**
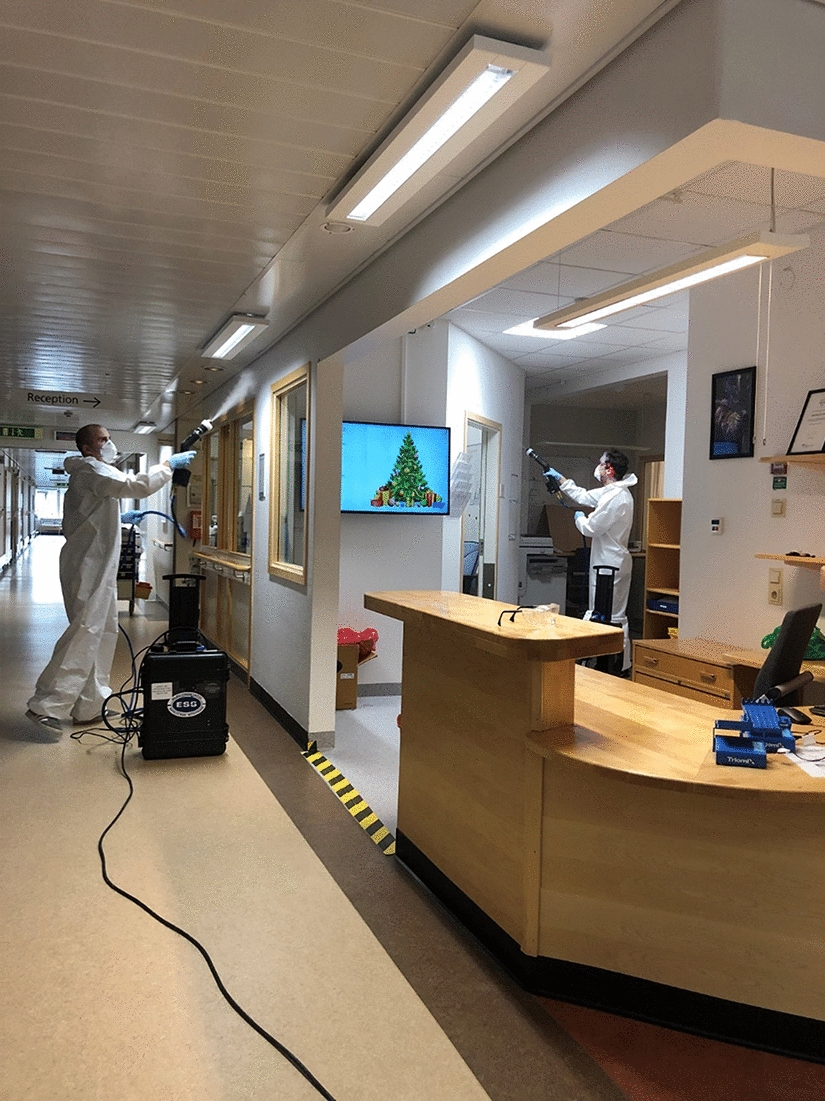
Treatment of the ward with the organic silicon compound using the electrostatic sprayers

The geriatric ward was chosen to be treated with Bioshield® 75. Both wards have an area of approximately 1000 m^2^ and all 12 patient rooms, common areas, kitchen, nurses’ offices, storage rooms and corridors were treated. All floors, walls and objects were treated with two coats of Bioshield® using two electrostatic sprayers (Fig. 1). All applications were monitored for quality control by the manufacturer’s representative. The application consumed approximately 20 L of the diluted compound and took 9 h over two and a half days to complete. The ward was in clinical use and the patients were moved to one end of the ward while treating the first rooms and then systematically moved back into the treated rooms while the work progressed. The initial treatment was followed by once-a-week treatment on either Tuesday, Wednesday or Thursday, when all high-touch areas were hand sprayed, consuming approximately 2 L of the same diluted compound each time.

The normal cleaning and hygiene routines were unchanged during the study period; they included the daily cleaning of floors and the alcohol treatment of high-touch areas. On the control ward, only the normal hygiene routines were performed.

The normal hand-hygiene for the hospital staff includes handwash when visibly dirty, after contact with patients who suffer vomiting and diarrhea. Alcohol hand disinfection application of 2–4 mL before and after close patient contact such as, catheterization, wound dressing, food handling, drug management and after every handwash.

### Hospital acquired infections (HAI)

All patients with a suspected or established infection were registered according to a specified protocol, including postoperative wound infection, septicemia, bacterial arthritis, pneumonia, urinary tract infection, influenza, gastroenteritis and upper respiratory tract infection. Patients with more than one infection were still registered as one infection. The registration was handled by two nurses on each ward. The first period of registration was from December 2017 until February 2018. The period was chosen because, historically, the winter season has seen serious outbreaks of calicivirus infections. After treatment, the second period of registration was from December 2018 until February 2019.

### Microbial methods

Twenty defined cultivation areas were chosen on each ward (total 40 culture locations), mostly high-touch areas (18/20), while 2/20 were from floors [9]. The locations of the cultures were blinded to the ward personnel and the single staff member performing the treatments of the high-touch areas. Examples of high-touch areas include handles, switches, computer keyboards, blood pressure cuffs, bed rails, toilet seats and water taps. Cultures were taken in the afternoon on the day before treatment and then after 1, 2, 4, 8, 12, 14 and 16 weeks. The cultures after treatment were obtained on Mondays (five occasions) or Tuesdays (two occasions) early in the morning before the daily routine cleaning. All cultures were obtained by the same biomedical analyst (LR).

### Bacterial environmental survey

A bacterial environmental culture was taken unannounced on both wards one month before the start of treatment to obtain an idea of the type of bacteria that could be expected. Depending partly on the result of the unannounced environmental culture before treatment and an ocular inspection of the wards, the hospital’s hygiene section decided that both wards would undergo so-called deep cleaning. It was performed the week before the start of treatment on the treated ward and during week 2 on the untreated ward. The environmental cultures were analyzed by the microbiologic laboratory at the hospital. The cultures were obtained using eSwab™ (pink cork) and covered an area of approximately 5 cm^2^. The bacteria which were cultured were one quantitative culture (skin flora), followed by specific agents, *staphylococcus aureus*, enterococci and gram negative rods. The samples were grown on blood plates, incubated in a thermostatically controlled room at 36 °C and read after 1 to 2 days, depending on the type of bacteria. The grading was given as no growth (0), sparse growth, 1–10 colonies cfu (colony forming units) (I), moderate growth, 10–100 cfu (II), abundant growth, > 100 cfu (III), or pathogen growth (III). Environmental cultures were repeated after 8, 12, 14 and 16 weeks as a quality control and as a complement to the Petrifilm™ plates.

### 3M™ Petrifilm™ plates

Based on the results of the bacterial environmental cultures, three different Petrifilm plates were used, the aerobic count plate for the enumeration of aerobic flora, the *E. coli* and coliform count plate and the staph express count system, for the enumeration of *Staphylococcus aureus*. The 3M™ Quick Swab Method and 4 mL of Letheen broth were used to neutralize any residual disinfectant. The area which was sampled was approximately 5 cm^2^. Using a pipette, 1 mL was drawn from the tube and dispensed onto the Petrifilm for the different plates. The plates were incubated in a heating cabinet at 34–35 °C and read after 1 to 2 days, depending on the type of bacteria, according to the manufacturer’s instructions. The results were given as the number of colonies on each plate. All the cultures were read and calculated by the same biomedical analyst. To obtain the number of cfu/cm^2^, the number of colonies must be recalculated with respect to surface area and dilution. In this study, the tested area was 5 cm^2^ and a 4 mL 3M Swab Sampler was used. As an example, if the number of colonies on the plate after incubation was 100, the result would be: 100 CFU × 4 mL = 400 CFU/5 cm^2^ or 80 CFU/cm^2^.

### Hygiene failures

Although not generally accepted, limit values have been proposed for a good cleaning standard in healthcare environments. For hand-touch sites, total aerobic colony counts (ACC) of < 2.5 to 5 CFU (colony forming units) per cm^2^ and < 1 CFU/cm^2^ for hospital pathogens (e.g. staph aureus) have been suggested as microbiologic benchmarks [10]. The breaking point for cleanliness using the Petrifilm, given the sample area and dilution in the present study, would be 6.25 CFU. There are no known defined good cleaning standards for environmental bacterial cultures, but it appears reasonable to apply 0 and sparse growth.

### Statistical methods

The primary variable in the study was the total amount of ACC in CFU. In the power analysis, it was estimated that the total aerobic colony counts would be 50% lower on the treated ward compared with the non-treated ward over the study period. To achieve a power of 80%, 15 cultures are required on each occasion from each ward, if the p-value is set at 0.05. To increase power, 2 × 20 cultures from the two wards were obtained together on every occasion. The total ACC is given as the total amount of CFUs and the number of HAIs is given in percent. The unpaired Student’s t-test was used for the comparison of CFUs between the wards and the non-parametric Mann–Whitney U test was used for the comparison of HAIs. More specifically the percentage of patients with an HAI in each ward was registered every day and a comparison between wards was calculated after the whole study period. In the results the mean percentage of HAI, range and median are reported.

## Results

**Fig. 2:**
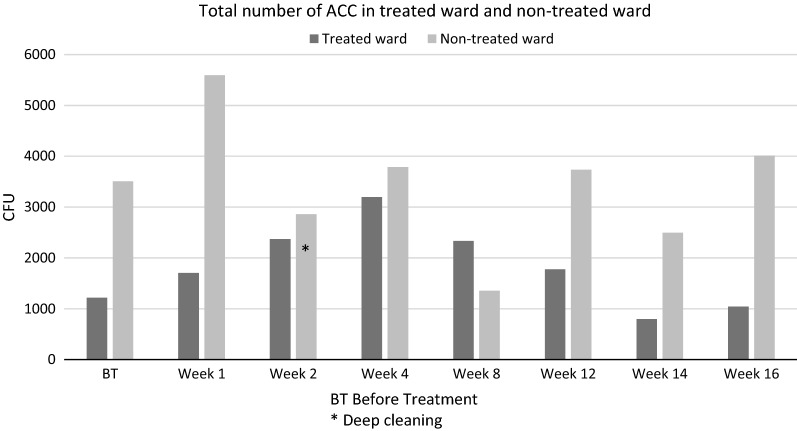
The total number of ACC found on twenty cultivation sites before treatment and during 4 months of follow up in the two wards. The treated ward had undergone deep cleaning just before the base line cultivation, while the non-treated ward underwent deep cleaning at week 2. The ratio of hygiene failures was 69% in treated ward and 81% in non-treated ward

The numbers of ACC before treatment and up to 16 weeks are reported in Fig. 2. The total ACCs were 47% lower on the treated ward compared with the non-treated ward over the study period (p = 0.02). The total number CFU of Staph aureus during the 4 months follow up period in the treated ward was 102, and in the untreated ward 58. For *E. coli* it was 0 and 146 in the treated and untreated ward respectively.

The first registration of HAI was performed during a three-month period between 1st of December 2017 and 28th of February 2018. The frequency of patients with HAI at the first registration on the AOG ward was 20.0% (0–50.0%), and the corresponding figures for the AO ward were 22.7% (4.5–43.8%), p = 0.5. The second registration of HAI after treatment was made between 3rd of December 2018 and 28th of February 2019. During that period, the frequency of HAI in the AOG was12.5% (0–30%), and the corresponding figures for the AO were 25.0% (4.0–47.6%), p = 0.0001, Table 1.

**Table 1:** Median HAI frequencies in percent at the first and second measurement period

Ward	2017/2018	2018/19
AO non-treated	22.7%	25.0%
AOG treated	20.0%	12.5%

**Fig. 3:**
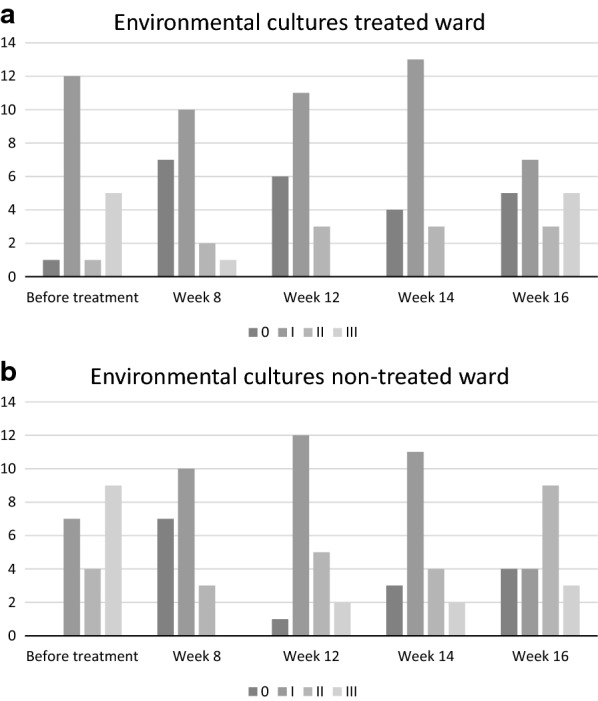
**a**, **b** Environmental cultures 4 weeks before treatment and during treatment, classified as no growth 0, sparse growth I, moderate growth II, abundant growth III. The ratio of hygiene failures during the treatment period was 21% in the treated ward and 35% in the non-treated ward

The results of the environmental cultures taken unannounced one month before treatment and in weeks 8, 12, 14 and 16 are reported in Fig. 3a and b. Hygiene failures during the second period were 26% in the treated ward and 35% in the non-treated ward.

Hygiene failures based on ACC were 69% in treated ward and 81% in non-treated ward.

## Discussion

The principal finding in this study was that, by using a long-lasting organosilane coating, the total aerobic colony counts could be reduced and the number of hospital acquired infections decreased.

There are a few people who question that there is a link between poor hygiene and the risk of infections [11–13]. Total ACC is a way to measure cleanliness. The total ACC at the 20 culture sites varied markedly in the present study, on both the untreated ward and the treated ward. The total aerobic colony counts on the treated ward was about half that on the non-treated ward over the study period. Using an organosilane-based compound, but also including an quarternary ammonium component, Tamimi et al. saw a 99% reduction in the total average bacterial count on all treated surfaces for at least eight weeks [8], while Boyce et al. were unable to find any sustained antimicrobial effect using two different organosilane products, in addition to routine cleaning with a quarternary ammonium-based disinfectant. Bacterial cultures were taken daily for four weeks [14]. Ellingson et al. reported a significant reduction in both HAI and the environmental bioburden using an antimicrobial surface coating. They saw a 36% decline in HAIs in treated hospitals and a total decline in bacterial numbers of 64% and 75% in the two treated hospitals during a 12-month period when comparing before and after application [15].

Some factors causing the variation in the effect of the long-lasting organosilane are probably cleaning procedures, standard hygiene routines among staff, patient mix and patient overcrowding during the study period. There are no regulatory requirements for checking hygienic standards on hospital wards and reference material is therefore missing. Unfortunately, more systematic bacterial environmental surveys are only performed in cases of serious outbreaks of infections to control disease transmission paths, but normal hygiene standards are only checked by ocular inspections.

Benchmarks for cleanliness in high-touch areas have been proposed, but they are not generally accepted [10, 16]. Using the proposed benchmarks in the present study, the majority of contact surfaces still remain a reservoir for the continued spread of bacteria on both wards, with 81% of high-touch sites on the untreated ward and 69% in the treated ward being hygiene failures, according to the Petrifilm cultures.

White et al. reported that 25% of the surfaces in a five-bed intensive care unit did not meet the hygiene standard [1]. Dancer et al. screened ten hand-touch sites on two surgical wards over two six-month periods and reported 64%, 62% and 44% failures in beds/hoists, bedside lockers and overbed tables respectively [17]. Using the environmental cultures and defining 0 growth and sparse growth as cleanliness appears to be more favorable in the present study, where hygiene failures were found in the samples from the treated ward in 26% and in the untreated ward in 35%.

The question of how to further improve and reduce the bioburden is important. The initial and repeated treatments with the long-lasting organosilane in the high-touch areas were not coordinated with alcohol swiping. Theoretically at least, it could be an advantage to spray organosilane onto a clean surface without remaining biofilm, after which the effect might improve.

During the study period, the standard cleaning procedures were followed. Today, the effect of cleaning is not routinely controlled by bacterial cultures or by adenosine triphosphate (ATP) bioluminescence assays. Paying more attention to the work of the cleaning staff, emphasizing the importance of good cleaning procedures and creating an opportunity to give positive and negative feedback could also have a favorable effect [18, 19].

The frequency of HAI in the present study was fairly high, but, when it came to total ACC, it varied substantially on both wards during the study period. The Swedish Association of Local Authorities and Regions (SALAR) is an employers’ organisation and represents and advocates for local governments in Sweden. It performs a point prevalence measurement for HAI every year. In 2019, 9.2% of the patients had an HAI based on a survey of 13,633 patients [20]. In a retrospective one-day survey of randomly selected inpatients, Magill et al. reported that 4% of 11,282 patients had one or more healthcare-associated infections [21]. The reason for the relatively high frequencies of HAI seen in the present study could be that many of the relatively old patients had undergone surgery due to fragility fractures, as well as for ischemic conditions. However, almost cutting the HAIs in half on the treated ward appears promising.

One of the reasons for conducting the study was to investigate whether organosilane treatment could prevent or reduce a calicivirus outbreak. Historically, the hospital has had yearly outbreaks of calicivirus infections during the winter season, which was the reason why the period between December and February was chosen to conduct the present study. However, during both registration periods, for unknown reasons, no such outbreaks were seen.

Due to the large variation in both total ACC and HAI during the study period, the promising results of the present study must be interpreted cautiously. Although the number of total ACC decreased significantly, most contact surfaces remained as possible sources of contamination. The property of an ideal future antimicrobial coating must be that treated high-touch areas remain non-contaminated over a longer time period than is possible to achieve using alcohol or peroxide products. Furthermore, they should be non-toxic for humans and not result in the development of resistance among microbes.

## Limitations

The initial environmental bacterial culture brought about a need for deep cleaning. For capacity reasons, it could only be implemented on one of the wards before the start of the study and it was followed on the other ward during week two. The deep cleaning probably had a bacteristatic effect for at least a few days [3]. The study length of three to four months is probably too short, given the large variation in bacterial growth and HAIs. It is not a limitation in the present study, but it is noteworthy that there are no routines for cleanliness standards in the hospital environment, considering the presence of multi-resistant bacteria and antibiotic resistance.

## Conclusions

The use of a long-lasting antimicrobial organosilane coating appears to reduce the bioburden and reduce HAI. Since the incidence of HAI varies substantially over time, longer observation times are needed.

## Supplementary information

**Additional file 1.** Raw data to Figs. 2 and 3.

## Data Availability

The datasets used and/or analysed during the current study are available from the corresponding author on reasonable request.
