# Supplementary material for: Antimicrobial coating is associated with significantly lower aerobic colony counts in high-touch areas in an orthopedic ward environment
Source: Ann Clin Microbiol Antimicrob. 2020 Dec 14;19:62. doi: 10.1186/s12941-020-00406-7 (PMC7737259; doi:10.1186/s12941-020-00406-7)
Supplement: Supplementary file 1 — Additional file 1. Raw data to Figs. 2 and 3. [file 12941_2020_406_MOESM1_ESM.docx]

| CS |  | BT |  |  | W 1 |  |  | W 2 |  |  | W 4 |  |  | W8 |  |  | W  12 |  |  | W  14 |  |  | W 16 |  |
| --- | --- | --- | --- | --- | --- | --- | --- | --- | --- | --- | --- | --- | --- | --- | --- | --- | --- | --- | --- | --- | --- | --- | --- | --- |
|  | S | E | A | S | E | A | S | E | A | S | E | A | S | E | A | S | E | A | S | E | A | S | E | A |
| 1 | *0* | *0* | *99* | *0* | *0* | *14* | *0* | *0* | *8* | **0** | **0** | **3** | *0* | *0* | *9* | *0* | *0* | *63* | *0* | *0* | *16* | *0* | *0* | *8* |
| 2 | *0* | *0* | *30* | *0* | *0* | *17* | *0* | *0* | *13* | *0* | *0* | *12* | *0* | *0* | *7* | *0* | *0* | *22* | **0** | **0** | **2** | **0** | **0** | **0** |
| 3 | *2* | *0* | *63* | *0* | *0* | *200* | *0* | *0* | *140* | *0* | *0* | *1000* | *0* | *0* | *32* | *0* | *0* | *800* | *0* | *0* | *27* | *0* | *0* | *30* |
| 4 | *3* | *0* | *720* | *0* | *0* | *23* | *2* | *0* | *6* | **0** | **0** | **3** | **0** | **0** | **0** | *0* | *0* | *11* | **0** | **0** | **4** | **0** | **0** | **5** |
| 5 | *15* | *0* | *106* | **0** | **0** | **3** | **0** | **0** | **1** | **0** | **0** | **0** | **0** | **0** | **6** | *0* | *0* | *81* | *5* | *0* | *15* | *0* | *0* | *740* |
| 6 | *0* | *0* | *42* | *0* | *0* | *9* | *0* | *0* | *13* | *0* | *0* | *160* | *0* | *0* | *8* | *0* | *0* | *8* | *0* | *0* | *21* | *0* | *0* | *20* |
| 7 | *0* | *0* | *80* | *0* | *0* | *1601* | *0* | *0* | *300* | *0* | *0* | *25* | *0* | *0* | *900* | *0* | *140* | *800* | *0* | *0* | *520* | *0* | *0* | *120* |
| 8 | *0* | *0* | *101* | *0* | *0* | *15* | *0* | *0* | *8* | *0* | *0* | *11* | **0** | **0** | **4** | *0* | *0* | *20* | *8* | *0* | *88* | *0* | *0* | *96* |
| 9 | *0* | *0* | *580* | *0* | *0* | *720* | *0* | *0* | *88* | *0* | *0* | *15* | **0** | **0** | **6** | *0* | *0* | *54* | *0* | *0* | *15* | *12* | *0* | *86* |
| 10 | *1* | *0* | *22* | *0* | *0* | *640* | *0* | *0* | *240* | *0* | *0* | *43* | *0* | *0* | *41* | *3* | *0* | *40* | *0* | *0* | *16* | *0* | *0* | *39* |
| 11 | *2* | *0* | *1100* | *0* | *0* | *32* | *0* | *0* | *180* | *0* | *0* | *420* | *0* | *0* | *10* | *0* | *0* | *45* | **0** | **0** | **4** | *0* | *0* | *38* |
| 12 | *0* | *0* | *220* | *0* | *4* | *280* | *0* | *0* | *400* | *0* | *0* | *1000* | *0* | *0* | *9* | *0* | *0* | *56* | *0* | *0* | *1200* | *0* | *0* | *1000* |
| 13 | *1* | *0* | *43* | *0* | *0* | *67* | *0* | *0* | *1200* | *0* | *0* | *1000* | *0* | *2* | *120* | *0* | *0* | *1000* | *0* | *0* | *420* | *0* | *0* | *120* |
| 14 | *0* | *0* | *17* | *0* | *0* | *1602* | *0* | *0* | *160* | *0* | *0* | *42* | *0* | *0* | *87* | *0* | *0* | *660* | *0* | *0* | *14* | *4* | *0* | *1600* |
| 15 | 0 | 0 | 77 | 0 | 0 | 50 | 0 | 0 | 16 | 0 | 0 | 9 | 0 | 0 | 6 | 0 | 0 | 8 | 0 | 0 | 39 | 0 | 0 | 38 |
| 16 | 0 | 0 | 23 | 0 | 0 | 240 | 0 | 0 | 5 | 0 | 0 | 3 | 0 | 0 | 14 | 0 | 0 | 5 | 0 | 0 | 0 | 0 | 0 | 3 |
| 17 | *0* | *0* | *150* | *0* | *0* | *60* | *0* | *0* | *12* | *0* | *0* | *30* | *0* | *0* | *14* | *0* | *0* | *41* | *0* | *0* | *10* | *0* | *0* | *8* |
| 18 | **0** | **0** | **1** | **0** | **0** | **0** | **0** | **0** | **6** | **0** | **0** | **2** | **0** | **0** | **0** | **0** | **0** | **0** | *0* | *0* | *29* | *0* | *0* | *15* |
| 19 | *0* | *0* | *7* | *0* | *0* | *12* | **0** | **0** | **0** | **0** | **0** | **3** | **0** | **0** | **0** | **0** | **0** | **0** | *0* | *0* | *9* | *0* | *0* | *22* |
| 20 | *0* | *0* | *26* | *0* | *0* | *10* | *0* | *0* | *64* | *0* | *0* | *7* | *0* | *0* | *62* | *0* | *0* | *21* | *0* | *0* | *47* | *0* | *0* | *25* |

Table 2 A. Numbers of CFUs in the non-treated ward, Cultivation Site (CS), Before Treatment (BT), *Staph Aureus* (S), *E-Coli* (E), Aerobic (A), Week (W), hygiene failures in italics (81%), clean in bold, floors in normal text

| CS |  | BT |  |  | W 1 |  |  | W 2 |  |  | W 4 |  |  | W8 |  |  | W  12 |  |  | W14 |  |  | W  16 |  |
| --- | --- | --- | --- | --- | --- | --- | --- | --- | --- | --- | --- | --- | --- | --- | --- | --- | --- | --- | --- | --- | --- | --- | --- | --- |
|  | S | E | A | S | E | A | S | E | A | S | E | A | S | E | A | S | E | A | S | E | A | S | E | A |
| 1 | *0* | *0* | *12* | *0* | *0* | *29* | *0* | *0* | *15* | *0* | *0* | *300* | *0* | *0* | *38* | **0** | **0** | **0** | *0* | *0* | *14* | **0** | **0** | **5** |
| 2 | **0** | **0** | **1** | **0** | **0** | **0** | *0* | *0* | *7* | **0** | **0** | **5** | **0** | **0** | **0** | **0** | **0** | **0** | **0** | **0** | **6** | **0** | **0** | **5** |
| 3 | *0* | *0* | *10* | *0* | *0* | *7* | *0* | *0* | *80* | *0* | *0* | *68* | **0** | **0** | **2** | *0* | *0* | *46* | *0* | *0* | *43* | *0* | *0* | *35* |
| 4 | *3* | *0* | *1* | **0** | **0** | **3** | *0* | *0* | *220* | **0** | **0** | **1** | *0* | *0* | *7* | *0* | *0* | *220* | **0** | **0** | **6** | *0* | *0* | *16* |
| 5 | *12* | *0* | *11* | *0* | *0* | *64* | *0* | *0* | *140* | *0* | *0* | *10* | *0* | *0* | *480* | *0* | *0* | *260* | *0* | *0* | *15* | *0* | *0* | *25* |
| 6 | *1* | *0* | *29* | *34* | *0* | *600* | *0* | *0* | *100* | *0* | *0* | *25* | *0* | *0* | *61* | **0** | **0** | **6** | *0* | *0* | *17* | **0** | **0** | **5** |
| 7 | *0* | *0* | *3* | *0* | *0* | *25* | *0* | *0* | *29* | *0* | *0* | *200* | *1* | *0* | *800* | *0* | *0* | *85* | *0* | *0* | *440* | *0* | *0* | *9* |
| 8 | *0* | *0* | *39* | *0* | *0* | *3* | *0* | *0* | *41* | *0* | *0* | *1200* | *0* | *0* | *20* | *0* | *0* | *480* | *0* | *0* | *7* | *0* | *0* | *140* |
| 9 | *0* | *0* | *1* | *0* | *0* | *3* | *0* | *0* | *41* | *0* | *0* | *65* | *0* | *0* | *12* | **0** | **0** | **4** | **0** | **0** | **0** | **0** | **0** | **6** |
| 10 | *0* | *0* | *52* | *0* | *0* | *30* | *0* | *0* | *53* | *0* | *0* | *140* | **0** | **0** | **5** | *0* | *0* | *28* | *0* | *0* | *23* | *0* | *0* | *8* |
| 11 | *0* | *0* | *11* | *0* | *0* | *9* | *0* | *0* | *140* | *0* | *0* | *180* | *0* | *0* | *83* | *0* | *0* | *12* | **0** | **0** | **0** | **0** | **0** | **0** |
| 12 | *0* | *0* | *620* | *0* | *0* | *480* | *0* | *0* | *200* | *0* | *0* | *400* | *0* | *0* | *500* | *0* | *0* | *54* | *0* | *0* | *7* | *0* | *0* | *360* |
| 13 | *0* | *0* | *224* | *0* | *0* | *77* | *0* | *0* | *48* | *0* | *0* | *280* | *0* | *0* | *200* | *0* | *0* | *41* | *0* | *0* | *12* | *0* | *0* | *48* |
| 14 | *0* | *0* | *0* | *0* | *0* | *160* | *0* | *0* | *1000* | *0* | *0* | *120* | *0* | *0* | *8* | *0* | *0* | *7* | **0** | **0** | **3** | **0** | **0** | **0** |
| 15 | 0 | 0 | 61 | 0 | 0 | 31 | 0 | 0 | 24 | 0 | 0 | 52 | 0 | 0 | 25 | 0 | 0 | 25 | 0 | 0 | 14 | 0 | 0 | 120 |
| 16 | 2 | 0 | 54 | 2 | 0 | 30 | 0 | 0 | 160 | 0 | 0 | 45 | 0 | 0 | 24 | 45 | 0 | 400 | 0 | 0 | 118 | 0 | 0 | 200 |
| 17 | *0* | *0* | *59* | *0* | *0* | *140* | *0* | *0* | *60* | *2* | *0* | *100* | *0* | *0* | *67* | *0* | *0* | *100* | *0* | *0* | *54* | *0* | *0* | *35* |
| 18 | *0* | *0* | *16* | *0* | *0* | *11* | *0* | *0* | *8* | **0** | **0** | **1** | **0** | **0** | **3** | **0** | **0** | **3** | *0* | *0* | *15* | *0* | *0* | *16* |
| 19 | **0** | **0** | **5** | **0** | **0** | **2** | *0* | *0* | *7* | **0** | **0** | **0** | **0** | **0** | **0** | **0** | **0** | **5** | **0** | **0** | **2** | *0* | *0* | *8* |
| 20 | *0* | *0* | *9* | **0** | **0** | **0** | **0** | **0** | **0** | **0** | **0** | **6** | **0** | **0** | **1** | **0** | **0** | **0** | **0** | **0** | **0** | **0** | **0** | **0** |

Table 2 B. Number of CFU in the treated Ward, Cultivation Site (CS), Before Treating (BT), *Staph Aureus* (S), *E-Coli* (E), Aerobic (A), Week (W), hygiene failures in italics (69%), clean in bold and floors in normal text

| Cultivation  site | -4 week | Week 8 | Week 12 | Week 14 | Week 16 |
| --- | --- | --- | --- | --- | --- |
| 1 | *III (SA)* | **I** | **I** | *II* | **I** |
| 2 | *III (SA)* | **0** | *II* | **0** | **0** |
| 3 | *II* | **0** | *III (E)* | *II* | *II* |
| 4 | **I** | **0** | **I** | **I** | **0** |
| 5 | *III (SA)* | **I** | *II* | **0** | *II* |
| 6 | **I** | **0** | **0** | **I** | **I** |
| 7 | *II* | *II* | *III (E)* | *III (E)* | **I** |
| 8 | **I** | **I** | **I** | *II* | *II* |
| 9 | *II* | **I** | **I** | *II* | *III (SA)* |
| 10 | *III (SA)* | **I** | **I** | **I** | *III* |
| 11 | *II* | **I** | **I** | **I** | *II* |
| 12 | *III (SA)* | *II* | *II* | *III (E)* | *II* |
| 13 | *III (SA)* | **I** | **I** | **I** | *II* |
| 14 | **I** | *II* | *II* | **I** | *II* |
| 15 | I | 0 | I | I | II |
| 16 | III (SA) | I | I | I | II |
| 17 | *III (SA)* | **I** | **I** | **I** | **0** |
| 18 | **I** | **0** | **I** | **I** | *III (SA)* |
| 19 | **I** | **0** | **I** | **0** | **0** |
| 20 | *III (SA)* | **I** | *II* | **I** | **I** |

Table 4 A. Environmental cultures in the non-treated ward, SA (*Staph aureus*), E (*Enterococci*), hygiene failures in italics (35%), clean in bold, and floors in normal text.

| Cultivation site | -4 week | Week 8 | Week 12 | Week 14 | Week 16 |
| --- | --- | --- | --- | --- | --- |
| 1 | **1** | **0** | **0** | **I** | *III (SA)* |
| 2 | *III (E)* | **0** | **I** | **I** | **I** |
| 3 | *I* | **I** | **0** | *II* | *III (SA)* |
| 4 | **I** | **0** | *II* | **I** | **0** |
| 5 | *II* | *III* | **I** | **I** | *II* |
| 6 | **0** | *II* | **I** | **I** | **I** |
| 7 | **I** | *III* | *II* | *II* | **I** |
| 8 | **I** | **I** | **I** | **I** | **I** |
| 9 | **I** | **I** | **0** | **0** | **0** |
| 10 | **I** | **I** | **0** | **I** | **0** |
| 11 | **I** | **I** | **0** | **I** | **I** |
| 12 | *III (SA)* | *II* | **I** | **0** | *III* |
| 13 | *II* | **I** | **I** | *II* | *III* |
| 14 | **I** | *III (E)* | *III* | **0** | **0** |
| 15 | III | 0 | I | I | I |
| 16 | III (SA) | 0 | I | I | II |
| 17 | *III (SA)* | *II* | *II* | **I** | *I* |
| 18 | **I** | **0** | **I** | **I** | *II* |
| 19 | **I** | **I** | **I** | **I** | *III(SA)* |
| 20 | **I** | **0** | **0** | **0** | **0** |

Table 4 B. Environmental cultures in the treated ward, SA (*Staph aureus*), E (*Enterococci*), hygiene failures in italics (26%), clean in bold, and floors in normal text.
